# Knowledge and Attitude Toward Geriatric Palliative Care among Health Professionals in Vietnam

**DOI:** 10.3390/ijerph16152656

**Published:** 2019-07-25

**Authors:** Huyen Thi Thanh Vu, Long Hoang Nguyen, Thanh Xuan Nguyen, Thu Thi Hoai Nguyen, Tam Ngoc Nguyen, Huong Thi Thu Nguyen, Anh Trung Nguyen, Thang Pham, Cuong Tat Nguyen, Bach Xuan Tran, Carl A. Latkin, Cyrus S. H. Ho, Roger C. M. Ho

**Affiliations:** 1 Gerontology department, Hanoi Medical University, Hanoi 100000, Vietnam; 2 Scientific Research Department, National Geriatric Hospital, Hanoi 100000, Vietnam; 3 Center of Excellence in Behavioral Medicine, Nguyen Tat Thanh University, Ho Chi Minh City 770000, Vietnam; 4 Dinh Tien Hoang Institute of Medicine, Hanoi 100000, Vietnam; 5 Institute for Global Health Innovations, Duy Tan University, Da Nang 550000, Vietnam; 6 Institute for Preventive Medicine and Public Health, Hanoi Medical University, Hanoi 100000, Vietnam; 7 Bloomberg School of Public Health, Johns Hopkins University, Baltimore, MD 21205, USA; 8 Department of Psychological Medicine, National University Hospital, Singapore 119074, Singapore; 9 Department of Psychological Medicine, Yong Loo Lin School of Medicine, National University of Singapore, Singapore 119228, Singapore; 10 Institute for Health Innovation and Technology (iHealthtech), National University of Singapore, Singapore 119077, Singapore

**Keywords:** palliative care, elderly, geriatric, PCKT, FATCOD, Vietnam

## Abstract

This study assessed the knowledge and attitude toward palliative care for the elderly among health professionals in a tertiary geriatric hospital in Vietnam and explored their determinants. Cross-sectional data were obtained on 161 geriatric health professionals at the National Geriatric Hospital. Modified-Palliative Care Knowledge Test and Frommelt Attitudes Toward Care of the Dying instruments were used to measure knowledge and attitude toward geriatric palliative care. As a result, 40.5% physicians and 74.2% nurses showed insufficient knowledge about geriatric palliative care (*p* < 0.05). The lowest score was for dyspnea, following by gastrointestinal and pain problems. No significant difference was found regarding the attitude between physicians and nurses (*p* > 0.05). Health professional category, age, and years of experience were found to be associated with knowledge about palliative care. Meanwhile, only knowledge score had correlations with total attitude score (Coef. = 0.2; 95%CI = 0.1–0.3), attitude toward patients (Coef. = 0.1; 95%CI = 0.0–0.1) and toward patients’ family (Coef. = 0.1; 95%CI = 0.0–0.1). This study highlights a significant knowledge gap and preferable attitude toward palliative care for the elderly among physicians and nurses in the geriatric hospital. Intensive training about geriatric palliative care, focusing on pain, dyspnea and gastrointestinal issue management, should be performed to ensure the quality of palliative care services, especially in nurses.

## 1. Introduction

Longer life expectancy, along with alterations of end-of-life morbidity, poses great challenges in ensuring health care access for the aging population [1]. A global report estimated that approximately 901 million individuals aged 60 or above lived in the world at the end of 2015, accounting for 12% of the global population [2]. This number is forecasted to reach 1.4 billion in 2030 and 3.2 billion in 2100 [2]. Increasing life span means that more people have to face frailty due to the aging process, which increases the burden of chronic morbidity (such as cardiovascular disease, cancer, diabetes or dementia), as well as functional and cognitive impairments [3,4]. Moreover, as the elderly suffer serious life-threatening chronic illnesses with a long dying phase, the need to establish a comprehensive geriatric approach to improve their quality of life and avoid any unnecessary suffering at the end of life is becoming increasingly important.

Palliative care has been confirmed to help deal with end-of-life chronic illnesses. The World Health Organization defines that “palliative care is an approach that improves the quality of life of patients and their families facing the problem associated with life-threatening illness, through the prevention and relief of suffering by means of early identification, impeccable assessment, treatment of pain and other problems-physical, psychosocial, and spiritual” [5]. Palliative care is recommended, and should be provided based on the needs of patients, particularly the most vulnerable populations, such as the older people [6]. Notably, the provision of palliative care heavily depends on the knowledge, attitude, and skills of physicians and nurses [7]. However, global studies underline the deficit preparedness of health professionals in delivering palliative care, which may be attributable to the lack of education as well as faculties’ attitude toward palliative care [8,9,10,11]. Therefore, understanding gaps in knowledge and attitude toward palliative care for the elderly is deemed important for developing appropriate interventions to improve the quality of health care.

In Vietnam, following the call of the World Health Organization to integrate palliative care into general health service, the Ministry of Health official launched the palliative care initiative in 2005. After that, the National Guidelines on Palliative care for Cancer and HIV/AIDS patients was issued by Vietnam’s Ministry of Health (MoH) in 2006 [12]. Since then, no guidelines for other diseases—as well as for specific vulnerable populations such as the elderly—were developed, although Vietnam is among the countries having the most rapid increase of an aging population in the world. An annual report from the Ministry of Health published in 2016 underlined the important role of palliative care in geriatric care, the current unmet need of older people and the necessity of policy development toward geriatric palliative care in Vietnam [13].

A previous study on oncology nurses in Vietnam showed a remarkably low level of knowledge regarding palliative care, particularly in pain and other symptom management as well as psychological and spiritual perspectives [14]. Despite this insufficient knowledge, another study conducted throughout Vietnam indicated no national training course for Vietnamese health professionals in palliative care, and very few studies about knowledge and attitudes in palliative care among this population have been published [15]. Thus, this study assessed knowledge and attitude toward palliative care for the elderly among health professionals in a tertiary geriatric hospital in Vietnam and explored their determinants. The findings of this study would be served as a baseline for further interventions to improve the geriatric palliative care in Vietnam.

## 2. Materials and Methods 

### 2.1. Study Design and Participants

Cross-sectional data on 161 physicians and nurses (including 124 nurses and 37 physicians) were collected using a self-administered questionnaire at the National Geriatric Hospital from June to July 2017. These participants were randomly selected from the list of physicians and nurses in the hospital by using a computer software. The eligible criteria for enrolling participants included: (1) worked at the National Geriatric Hospital with full-time employment during the study period; (2) were willing to participate in the study and gave informed consent. The response rate was 100%. This study was approved by the Department of Geriatric, Hanoi Medical University, and National Geriatric Hospital (Code 1356/IRB).

### 2.2. Measurement

A self-administrated questionnaire was developed and piloted among 10 health professionals. Data of these participants were not included in the final dataset. After being invited by the research team, health professionals went to a private room and filled the questionnaire. The questionnaire did not include any questions that could disclose the identity of participants in order to ensure their confidentiality.

The questionnaire consisted of three sections: (1) socio-demographic and professionals characteristics; (2) knowledge about geriatric palliative care; and (3) attitude toward geriatric palliative care. The first part included information about gender, age, education level, years of working experience, frequency of treating or taking care terminally ill patients and previous education about palliative care. In terms of knowledge, we adopted the Palliative Care Knowledge Test (PCKT) [16]. In this study, based on geriatric expert’s review and opinion, we excluded three items that might not be practical in geriatric care in Vietnam (item “when cancer pain is mild, pentazocine should be used more often than opioid?”, item “the effects of opioids should decrease when pentazocine or buprenophine hydrochloride is used together after opioids are used?”, and item “morphine should be used to relieve dyspnea in cancer patients). We employed 17 items of this instrument and added five new items, which were selected via literature review and the opinion of experts regarding patients and health professionals’ needs for palliative care [17]. These items included:

(1) Terminally ill patients have the right to choose “do not resuscitate” (DNR) when his/her heart or breathing has stopped due to terminally ill disease condition and have no curative treatments available. (Philosophy of palliative care dimension) [18].
(2) Morphine relieves all kinds of pain. (Pain dimension) [19].
(3) Use of opioids can cause respiratory depression. (Psychiatric problems dimension) [17].
(4) Individuals who are taking opioids should also follow a bowel regime or use laxative to prevent constipation. (Gastrointestinal problems) [20].
(5) Opioid-induced nausea and/or vomiting occur in 80% or more of patients taking opioids (Gastrointestinal problems) [17].

There were 22 items used to measure physicians and nurses’ knowledge about geriatric palliative care in five dimensions: philosophy of palliative care (three items), pain (five items), dyspnea (five items), psychiatric problems (three items), and gastrointestinal problems (six items). Each item has three responses; “true”, “false” and “do not know”. Each correct answer was scored one point, while an incorrect or “do not know” answer was scored zero. The knowledge score ranged from 0 to 22, where a higher score indicated a higher level of knowledge. This scoring system was adopted from the original version of PCKT [16]. The level of health professionals’ knowledge was classified as follow: (1) insufficient (knowledge ≤ 50% of total score); (2) good (knowledge = 51–75% of total score); and (3) excellent (knowledge > 75% of total score). Cronbach’s alpha was 0.65.

Attitude toward geriatric palliative care was measured by using the Frommelt Attitudes Toward Care of the Dying (FATCOD) [21]. Originally, this tool contains 30 items (15 items with contents about positive attitude and 15 items with contents about negative attitude) using a five-point Likert scale. In this study, we employed 28 items (15 positive-attitude items and 13 negative-attitude items) with a score ranging from 28 to 140. We excluded two items; “giving care to the dying person is a worthwhile experience” and “there are times when death is welcomed by the dying person”, which might not be appropriate for the Vietnamese culture according to the experts’ opinion. We scored each item based on the guideline of the FATCOD [21]. Attitude toward patients was measured in 18 items (score ranging from 18 to 90) and attitude toward patients’ family was assessed in 10 items (score ranging from 10 to 50). Higher score reveals a more positive attitude toward palliative care [21]. The level of attitude was categorized as follow: (1) positive (score ≥ 75% of total score); (2) neutral (score = 50%–<75% of total score); and (3) negative (score < 50% of total score). The Cronbach’s alpha of this instrument was 0.75.

In this study, we applied the guidelines of the World Health Organization in translation and adaptation of research instrument [22] to translate the PCKT and FATCOD instruments into Vietnamese. Two language experts involved in translating these tools. Steps including forward-translation, expert panel discussion and back-translation [22] were conducted to ensure the quality of translation. After receiving feedback from participants in the pilot study, a substantial revision was conducted—including removing two items in original PCKT and FATCOD instruments—to ensure the cultural and logical perspectives of the questionnaire. The final version was approved by the principal investigators and leaders of the hospital.

### 2.3. Statistical Analysis

SPSS software version 20.0 was used to analyze data. Chi-squared and student-*t* tests were employed to measure the difference of knowledge and attitude between physicians and nurses, good knowledge and above or not, and positive attitude or not. The knowledge scores were transformed to a 100-point scale for increasing the comparability among dimensions. Multivariable Logistic and Tobit regression models were used to identify associated factors with knowledge and attitude toward geriatric palliative care. A *p*-value of less than 0.05 was considered the statistical significance. 

## 3. Results

Table 1 showed that the respondents consisted of 124 (77%) nurses and 37 (23.0%) physicians. The mean age and years of working experience were 29.6 (95% Confident interval (CI) = 28.7–30.6) and 6.2 (95%CI = 5.4–7.0) years. The majority of samples were females (76.4%). Most physicians had post-graduate education (78.4%), while most of the nurses had vocational training degree (42.2%). There were 39.1% of participants taking care of patients at the latest stage of any diseases daily, and 72.7% receiving training about palliative care previously.

Table 2 reveals that 35.1% physicians and 74.2% nurses had insufficient knowledge regarding geriatric palliative care (*p* < 0.05). The lowest score was for dyspnea, following by gastrointestinal and pain problems. Physicians had a significantly higher score of knowledge in 4/5 dimensions (philosophy of palliative care, pain, dyspnea, and psychiatric problems) and total scores compared to nurses (*p* < 0.05). Meanwhile, most of health professionals had neutral (79.5%) or positive (19.9%) scores of knowledge toward geriatric palliative care. No significant difference was found regarding the attitude between physicians and nurses (*p* > 0.05).

In Table 3, the rate of good knowledge or above was significantly higher in physicians, people aged >40 years, those having post-graduate degree, and having >10 years of experience (*p* < 0.05) compared to other groups. Also, the significant differences in positive attitude were found only in professional groups and education level (*p* < 0.05).

Table 4 shows associated factors with total knowledge and knowledge score in each dimension. Only age variable showed to correlate with the total knowledge score. Nurses had significantly lower score in pain dimension (Coef. = −19.5; 95%CI=−36.3–−2.7) compared to physicians. Those with 5–10 years of experience had a significantly lower score in psychiatric problems than that of those with less than five years of experience (Coef. = −16.5; 95%CI = −31.7–−1.4).

Meanwhile, in Table 5, multivariable regression models indicated that only knowledge score had correlations with total attitude score, attitude toward patients, and patients’ family. Specifically, increasing knowledge score would lead to increase total attitude score (Coef. = 0.2; 95%CI = 0.1–0.3), attitude toward patient score (Coef. = 0.1; 95%CI = 0.0–0.1) and attitude toward patient’s family score (Coef. = 0.1; 95%CI = 0.0–0.1).

## 4. Discussion

The current study reveals preliminary results about the knowledge and attitude of Vietnamese health professionals about geriatric palliative care, which potentially serves as a baseline for further interventions. The finding suggested a dramatic knowledge gap about palliative care for the elderly among geriatric physicians and nurses. This finding was in line with previous works in Ethiopia, which indicated that only 69.5% of nurses had inadequate knowledge [8], but lower in the study among Thai physicians (55.7%) and Indian nurses (89.0%) [9]. We supposed that the possible reasons for this might be a lack of experience regarding palliative care in our sample, or the length of training was not enough to provide sufficient information about palliative care. Of note, 72.7% of our health professionals have not yet received palliative care training, even those with a postgraduate degree, resulting in the limited knowledge of end-of-life care among our participants, which aligned with previous work [10]. 

In particular, the highest score was for the philosophy of palliative care aspect, which aligned with a previous study in Japan [23]. It might be because this aspect mentions a fundamental knowledge of palliative care, including definition, target population, and how palliative care should be delivered, which can be easy to remember. Meanwhile, we observed remarkably low scores in pain, dyspnea and gastrointestinal aspects, which was consistent with prior works [23,24]. This might be justified by a lack of training in using opioids appropriately in pain and dyspnea management, as well as the unavailability of opioids in Vietnam. A previous study in Vietnam showed that only 5% of health care workers reported the presence of oral morphine, and it is was rarely used—even if available [25]. Moreover, to date, there has not been an official guideline for opioid use in Vietnam, especially in geriatric palliative care, which might also reduce the awareness of proper opioids use in our participants. A comprehensive policy for opioids use is therefore needed to improve the awareness and knowledge of health professionals and facilitate palliative care for older people in the hospital. 

As expected, the level of palliative care knowledge of physicians was far higher than nurses. This might be explained by educational level, receiving palliative care training, as well as differences of clinical roles and responsibilities in care [26]. However, as nurses are individuals with direct and frequent communication with the elderly patients, suggesting palliative care knowledge is critically important for nurses and relevant directly to their jobs [27]. Therefore, this finding indicates an urgent need for palliative care training for both health professionals, especially nurses.

In addition to the low level of knowledge, most of participants had neutral (79.5%) and positive (19.9%) attitudes toward palliative care. These findings were aligned with studies in other settings, such as Turkey [28], Japan [29], India [9], and Ethiopia [8], which might be due to the same experience in caring dying patient, that participants had to take care terminally ill patients and give end of life care for the dying patients. The dominance of “neutral” attitude might be justified by insufficient knowledge and awareness of geriatric palliative care in our participants. Therefore, their answers tended to be balance between positive and negative attitudes. In fact, we found that knowledge and attitude toward palliative care had a significantly positive relationship, meaning that higher knowledge could lead to a more positive attitude. This result was consistent with previous studies in Saudi Arabia [30] and Vietnam [14], highlighting the need of comprehensive training to increase knowledge and change the attitude toward palliative care among our geriatric health professionals. 

This study suggests several implications. First, the training program should be designed and carried out to geriatric health professionals to promote the knowledge, attitude, and practice about palliative care for the elderly. Second, the training contents should especially focus on the significant knowledge gaps about dyspnea, gastrointestinal, and pain management. Third, educational interventions should be intensively provided to nurses, who play a critical role in palliative care as communicating closely with patients and patients’ family. Moreover, although health professionals who had a longer duration of working had a lower score in psychiatric problems, every health professionals should be recommended to take continuous training in order to enhance their current knowledge about palliative care.

Limitations of this study included a small sample size in a tertiary geriatric hospital, which reduced our ability to detect the associated factors with knowledge and attitude toward palliative care for the elderly. Additionally, this limitation decreases our generalizability, implying that we should be cautious when applying our findings to other settings. Moreover, despite using a simple random sampling method, it is possible that we did not include health professionals who had experience in palliative care. Also, the PCKT and FATCOD instruments used in this study cover several aspects of palliative care comprising philosophy, pain, dyspnea, psychiatric and gastrointestinal problems, as well as patients and patients’ family perspectives. Other components, such as communication or care planning, were not mentioned. This gap should be filled in future studies to comprehensively understand the knowledge and practice of health professionals toward geriatric palliative care. Final, using a self-administered questionnaire might induce bias, where the participants might see the correct answers, which possibly overestimate the level of knowledge of respondents.

## 5. Conclusions

This study highlights a significant knowledge gap and preferable attitude toward palliative care for the elderly among physicians and nurses in the geriatric hospital. Intensive training regarding geriatric palliative care, focusing on pain, dyspnea and gastrointestinal issue management, should be performed to ensure the quality of palliative care services, especially in nurses.

## Figures and Tables

**Table 1:** Characteristics of participants.

Characteristics	Physicians		Nurses		Total		*p*-Value
	*n*	%	*n*	%	*n*	%	
Total	37	23.0	124	77.0	161	100.0	
Gender							
• Male	14	37.8	24	19.4	38	23.6	0.02
• Female	23	62.2	100	80.7	123	76.4	
Education							
• Vocational training	0	0.0	68	54.8	68	42.2	<0.01
• College	0	0.0	34	27.4	34	21.1	
• Graduate	8	21.6	22	17.7	30	18.6	
• Post-graduate	29	78.4	0	0.0	29	18.0	
Frequency of taking care of patients at the latest stage of any diseases							
• Daily	16	43.2	47	37.9	63	39.1	0.01
• Weekly	9	24.3	10	8.1	19	11.8	
• Monthly	3	8.1	24	19.4	27	16.8	
• Few times per year	9	24.3	30	24.2	39	24.2	
• Never	0	0.0	13	10.5	13	8.1	
Had received training in palliative care							
• Yes	14	37.8	30	24.2	44	27.3	0.1
• No	23	62.2	94	75.8	117	72.7	
	Mean	95%CI	Mean	95%CI	Mean	95%CI	
Age	35.8	33.4–38.2	27.8	27.1–28.5	29.6	28.7–30.6	<0.01
Years of experience	10.4	7.9–12.8	5.0	4.3–5.6	6.2	5.4–7.0	<0.01

Abbrev: CI: Confident Interval.

**Table 2:** Knowledge and attitude toward palliative care among health professionals.

Characteristics	Physicians		Nurses		Total		*p*-Value
	*n*	%	*n*	%	*n*	%	
Knowledge about palliative care							
• Insufficient	13	35.1	92	74.2	105	65.2	<0.01
• Good	22	59.5	32	25.8	54	33.5	
• Excellence	2	5.4	0	0.0	2	1.2	
Attitude							
• Negative	1	2.7	0	0.0	1	0.6	0.02
• Neutral	24	64.9	104	83.9	128	79.5	
• Positive	12	32.4	20	16.1	32	19.9	
Characteristics	Mean	SD	Mean	SD	Mean	SD	
Knowledge domains (0–100)							
• Philosophy of palliative care	77.5	27.3	65.1	30.0	67.9	29.8	0.02
• Pain	62.2	23.5	44.2	18.8	48.3	21.3	<0.01
• Dyspnea	40.5	24.3	23.9	22.2	27.7	23.7	<0.01
• Psychiatric problems	77.5	30.5	47.0	30.7	54.0	30.7	<0.01
• Gastrointestinal problems	47.3	21.3	39.8	20.5	41.5	20.8	0.09
• Total score	57.4	13.4	41.6	13.6	45.2	15.1	<0.01
Attitude domains							
• Patients (18–90)	62.0	8.6	60.6	5.7	60.9	6.5	0.14
• Patients’ family (10–50)	37.2	5.5	37.3	3.3	37.3	3.9	0.31
• Total score (28–140)	99.2	12.9	97.9	7.4	98.2	9.0	0,14

Abbrev: SD: Standard Deviation.

**Table 3:** Knowledge and attitude toward palliative care according to different characteristics.

Characteristics	Good Knowledge or Above				*p*-Value	Positive Attitude				*p*-Value
	No		Yes			No		Yes		
	*n*	%	*n*	%		*n*	%	*n*	%	
Professional										
• Physicians	13	35.1	24	64.9	<0.01	25	67.6	12	32.4	0.03
• Nurse	92	74.2	32	25.8		104	83.9	20	16.1	
Gender										
• Male	23	60.5	15	39.5	0.49	32	84.2	6	15.8	0.47
• Female	82	66.7	41	33.3		97	78.9	26	21.1	
Age groups										
• 20–29	71	74.0	25	26.0	<0.01	81	84.4	15	15.6	0.21
• 30–39	31	58.5	22	41.5		40	75.5	13	24.5	
• >40	3	25.0	9	75.0		8	66.7	4	33.3	
Education										
• Vocational training	51	75.0	17	25.0	<0.01	56	82.4	12	17.7	0.01
• College	25	73.5	9	26.5		30	88.2	4	11.8	
• Undergraduate	20	66.7	10	33.3		26	86.7	4	13.3	
• Post-graduate	9	31.0	20	69.0		17	58.6	12	41.4	
Frequency of taking care patients at the latest stage of any diseases										
• Daily	39	61.9	24	38.1	0.09	49	77.8	14	22.2	0.7
• Weekly	9	47.4	10	52.6		14	73.7	5	26.3	
• Monthly	17	63.0	10	37.0		24	88.9	3	11.1	
• Few times per year	28	71.8	11	28.2		32	82.1	7	18.0	
• Never	12	92.3	1	7.7		10	76.9	3	23.1	
Had received training in palliative care										
• Yes	25	56.8	19	43.2	0.17	33	75.0	11	25.0	0.32
• No	80	68.4	37	31.6		96	82.1	21	18.0	
Years of experiences										
• <5 years	55	72.4	21	27.6	0.04	61	80.3	15	19.7	0.20
• 5–10 years	39	65.0	21	35.0		51	85.0	9	15.0	
• >10 years	11	44.0	14	56.0		17	68.0	8	32.0	

**Table 4:** Associated factors with knowledge about palliative care.

Characteristics	Good Knowledge or Above (1: Yes/0: No)		Total Knowledge Score (0–100)		Philosophy of Palliative Care Score (0–100)		Pain Score (0–100)		Dyspnea Score (0–100)		Psychiatric Problems Score (0–100)		Gastrointestinal Problems Score (0–100)	
	OR	95%CI	Coef.	95%CI	Coef.	95%CI	Coef.	95%CI	Coef.	95%CI	Coef.	95%CI	Coef.	95%CI
Professional														
Physician	ref		ref		ref		ref		ref		ref		ref	
Nurse	0.4	0.1; 2.2	−7.7	−18.8; 3.4	−9.0	−34.2; 16.3	−19.5 **	−36.3; −2.7	−9.7	−34.2; 14.9	−1.0	−29.7; 27.7	−3.7	−21.8; 14.4
Gender														
Male	ref		ref		ref		ref		ref		ref		ref	
Female	1.2	0.5; 3.0	1.8	−3.3; 6.9	2.6	−9.1; 14.2	3.0	−4.7; 10.8	−1.8	−13.3; 9.7	−3.2	−16.6; 10.1	4.8	−3.5; 13.2
Education														
Vocational training	ref		ref		ref		ref		ref		ref		ref	
College	1.1	0.4; 3.0	0.2	−5.3; 5.7	2.8	−9.8; 15.4	−2.2	−10.6; 6.1	4.8	−7.6; 17.3	−0.1	−14.7; 14.5	−2.2	−11.3; 6.8
Graduate	0.8	0.2; 2.6	4.9	−1.8; 11.6	9.3	−6.0; 24.7	3.6	−6.6; 13.7	0.8	−14.7; 16.3	14.4	−3.0; 31.9	3.0	−8.0; 13.9
Post-graduate	1.4	0.2; 12.4	6.2	−6.9; 19.4	1.9	−28.2; 32.0	−5.4	−25.4; 14.6	11.4	−18.0; 40.7	28.7 *	−5.5; 62.9	1.8	−19.7; 23.3
Age groups														
20–29	ref		ref		ref		ref		ref		ref		ref	
30–39	1.4	0.4; 4.4	6.7 **	0.1; 13.4	11.2	−4.1; 26.4	2.2	−7.9; 12.3	8.9	−6.3; 24.1	12.1	−5.4; 29.6	8.8	−2.1; 19.7
>40	1.1	0.1; 13.2	7.4	−7.6; 22.4	−7.5	−41.8; 26.8	12.7	−10.1; 35.5	12.4	−20.9; 45.8	24.2	−14.8; 63.2	5.8	−18.7; 30.4
Frequency of taking care patients at the latest stage of any diseases														
Daily	ref		ref		ref		ref		ref		ref		ref	
Weekly	1.5	0.5; 4.8	−0.3	−7.3; 6.7	−4.1	−20.1; 11.9	−3.9	−14.5; 6.8	6.0	−9.6; 21.6	4.0	−14.2; 22.3	−1.5	−13.0; 9.9
Monthly	1.2	0.4; 3.4	0.4	−5.7; 6.5	5.0	−8.9; 18.9	−4.1	−13.3; 5.1	6.0	−7.8; 19.8	−6.8	−22.8; 9.1	1.6	−8.3; 11.5
Few times per year	0.6	0.2; 1.6	−1.3	−6.7; 4.1	−2.2	−14.6; 10.1	−3.0	−11.2; 5.2	3.5	−8.8; 15.8	3.1	−11.1; 17.3	−5.1	−13.9; 3.8
Never	0.2	0.0; 1.8	−1.7	−9.8; 6.3	0.6	−17.8; 19.1	−4.2	−16.4; 8.1	4.0	−14.2; 22.2	−8.2	−29.6; 13.1	0.3	−12.9; 13.5
Had received training about palliative care														
Yes	ref		ref		ref		ref		ref		ref		ref	
No	0.7	0.3; 1.7	0.2	−4.5; 5.0	−0.2	−11.1; 10.6	−0.1	−7.3; 7.1	5.1	−5.8; 15.9	1.5	−10.9; 13.9	−2.1	−9.8; 5.7
Years of experiences														
<5 years	ref		ref		ref		ref		ref		ref		ref	
5–10 years	0.9	0.3; 2.7	−5.7 *	−11.4; 0.1	−2.6	−15.7; 10.5	−6.1	−14.8; 2.6	−10.0	−23.2; 3.1	−16.5 **	−31.7; −1.4	−3.0	−12.4; 6.4
11–15 years	1.0	0.2; 5.4	−2.6	−12.2; 6.9	8.7	−13.1; 30.5	0.7	−13.8; 15.1	−9.2	−30.8; 12.3	−10.6	−35.5; 14.3	−6.3	−21.9; 9.3
>16 years	6.9	0.2; 194.4	1.3	−16.1; 18.7	14.6	−25.1; 54.3	−9.4	−35.7; 17.0	6.2	−32.2; 44.6	−11.8	−57.0; 33.4	1.6	−26.8; 30.0

* *p* < 0.1; ** *p* < 0.05; *** *p* < 0.01.

**Table 5:** Associated factors with attitude toward palliative care.

Characteristics	Attitude Score (28–140)		Attitude Toward Patients Score (18–90)		Attitude Toward Patients’ Family Score (10–50)	
	Coef.	95%CI	Coef.	95%CI	Coef.	95%CI
Professional						
Physician						
Nurse	5.8	−1.3; 13.0	4.6 *	−0.6; 9.7	1.3	−1.9; 4.5
Gender						
Male						
Female	−1.9	−5.2; 1.4	−1.3	−3.7; 1.0	−0.6	−2.0; 0.9
Education						
Vocational training						
College	−0.9	−4.4; 2.6	−0.3	−2.8; 2.3	−0.6	−2.2; 1.0
Graduate	0.4	−3.9; 4.7	0.5	−2.7; 3.6	−0.0	−2.0; 1.9
Post–graduate	4.3	−4.2; 12.8	3.7	−2.4; 9.8	0.6	−3.2; 4.4
Age groups						
20–29						
30–39	0.4	−4.0; 4.7	1.0	−2.1; 4.1	−0.6	−2.6; 1.3
>40	7.3	−2.4; 17.0	5.4	−1.5; 12.4	1.9	−2.5; 6.2
Frequency of taking care of patients at the latest stage of any diseases						
Daily						
Weekly	−3.4 *	−7.3; 0.5	−2.5 *	−5.3; 0.3	−0.9	−2.7; 0.8
Monthly	−0.6	−4.0; 2.9	0.1	−2.4; 2.6	−0.7	−2.2; 0.9
Few times per year	3.0	−2.2; 8.2	2.3	−1.4; 6.1	0.7	−1.7; 3.0
Never	−3.4 *	−7.3; 0.5	−2.5 *	−5.3; 0.3	−0.9	−2.7; 0.8
Had received training about palliative care						
Yes						
No	−1.4	−4.4; 1.6	−1.7	−3.9; 0.5	0.3	−1.0; 1.7
Years of experiences						
<5 years						
5–10 years	−0.1	−3.8; 3.6	−0.5	−3.2; 2.1	0.4	−1.3; 2.1
11–15 years	−4.8	−10.9; 1.3	−3.2	−7.6; 1.2	−1.6	−4.3; 1.2
>16 years	−4.4	−15.5; 6.8	−3.2	−11.3; 4.8	−1.2	−6.2; 3.9
Total knowledge score	0.2 ***	0.1; 0.3	0.1 ***	0.0; 0.2	0.1 **	0.0; 0.1

* *p* < 0.1; ** *p* < 0.05; *** *p* < 0.01; Abbreviations: Coef.: coefficient; CI: confident interval.
